# Medical Reform

**Published:** 1847-08

**Authors:** 


### ARTICLE X.

### MEDICAL REFORM.

Professor Annan, of Lexington, Kentucky, a member of the late National Medical Convention, is out in the Western Lancet, against that body, in an article which has received the notice of many of our cotemporaries. The Annalist is particularly severe on it.

The sentiment that the community must be elevated before it is worth while for the physician to be educated thoroughly, because he will necessarily fall back to their level strikes us as most remarkable.

The objection to the recommendation of *Clinical Instruction*, because it is without the power of many of the schools to impart it, we think might bear on the teaching of many things universally regarded as important, with equal force, for many schools are quite defective. The following from a Philadelphia Journal is forcible and to the point:

"It is respectfully submitted that a school which proposes to educate *practitioners* of medicine, and cannot illustrate pathology with living cases, is bound either to close its doors or to announce that patients who employ its graduates must do so at their proper peril, regarding the title of doctor conferred by it as indicating no competency to treat disease."

But what surprises us most is that the editor of the Western Lancet should propose that Professor Annan's article gives the sentiments of the profession in the west. We are much mistaken if he is not. E.

